# The association between indicators of health and housing in people with Parkinson’s disease

**DOI:** 10.1186/s12877-016-0319-x

**Published:** 2016-07-27

**Authors:** Maria H. Nilsson, Susann Ullén, Henrik Ekström, Susanne Iwarsson

**Affiliations:** 1 Department of Health Sciences, Lund University, Lund, Sweden; 2 Memory Clinic, Skåne University Hospital, Malmö, Sweden; 3 R & D Centre, Skåne University Hospital, Lund, Sweden

**Keywords:** Activities of daily living, Canonical correlations, External control, Health, Housing, Parkinson’s disease

## Abstract

**Background:**

There are knowledge gaps about the life situation for people ageing with Parkinson’s disease (PD), with virtually no understanding of home and health dynamics. Therefore, the aim of the present study was to explore the association between aspects of health and objective as well as perceived housing in people with PD.

**Methods:**

Participants were recruited from three hospitals in the region of Skåne in southern Sweden. The sample for the present study included 231 (62 % men) participants with PD, with a mean age of 75 (min-max, 45–93) years. The data collection procedure included a self-administered postal survey and a subsequent home visit where structured interviews, observations and clinical assessments were administered. To study the association between aspects of health and housing canonical correlation was applied. Twelve variables (6 in the health and 6 in the housing set) were included. This corresponds to about 20 individuals per variable and is considered sufficient to accurately interpret the largest (i.e., first) canonical correlation.

**Results:**

The analysis between the health variables and housing variables set yielded two significant pairs of variates with the canonical correlations 0.68 (*p* < 0.0001) and 0.33 (*p* = 0.0112), respectively. For the first pair of variates the canonical R^2^ was 0.46. The results showed that external control beliefs and behavioral aspects of meaning of home contributed the most to the housing variate, whereas difficulties/dependence in activities of daily living (ADL) and functional limitations contributed the most to the health variate. Although a significant relationship was found for the second canonical correlation, the shared variance between the two variates was considerably lower; R^2^ = 0.11.

**Conclusions:**

This study suggests that people with PD who have more functional limitations, difficulties in ADL and are more dependent perceive their homes as less meaningful from a behavioral perspective. Moreover, they tend to rely on external influences managing their housing situation. With this kind of knowledge at hand, health care and social services professionals are in a better position to observe and efficiently address problems related to health and housing among people with PD.

## Background

With an increased life expectancy for the general population as well as for people ageing with chronic diseases [1] there are major challenges not only to individuals and their families but also to health care and societal planning [2]. For example, an increasing proportion of older people remain living in their ordinary homes despite health decline and disability [3]. Major gerontology studies typically involve general population samples with little attention to sub-groups with specific diagnoses. Consequently, little is known about the complex relationships of housing and health in different sub-groups of the ageing population, such as people with Parkinson’s disease (PD).

In terms of theory, gerontology studies that target person-environment relations most often refer to Lawton’s Ecological Theory of Ageing (ETA) [4, 5]. Herein the person is defined in terms of a set of competencies and the environment is defined in terms of press. When health declines in very old age, the environmental pressure (E) often exceeds the personal (P) capacities, resulting in more person-environment (P-E) fit problems, maladaptive behavior and negative health outcomes. Research on health and housing in very old age has considered contextual factors such as objective and perceived aspects of housing [6–8]. As to objective aspects of housing, potential physical environmental barriers can be assessed in relation to whether they comply with public standards and guidelines for built environment design. Perceived aspects of housing relate to health in very old age [6–8] and include the following constructs: usability, housing satisfaction, meaning of home, and housing-related control beliefs [9]. A previous study by Oswald et al. used canonical correlations in order to study the relationships between health and housing in five European countries. It involved very old single community-living people; the results indicated that very old people who perceive their home as meaningful and useful, and who think that external influences are not responsible for their housing situation are more independent in daily activities and have a better sense of well-being [8]. To date, such relationships have not been studied with other sub-groups of the ageing population and with little attention to specific diagnoses.

Ageing with a progressive and chronic disease such as PD imposes specific challenges. Already early on during the disease course, the performance of activities of daily living (ADL) is affected [10, 11]. People with PD are more likely to move to assisted living facilities and at an earlier age than people in general [12], and falls are among the leading reasons for residential care admittance [13]. This causes high costs to society [12] and has large consequences for those affected.

In two explorative studies [14, 15] we found that very old people with self-reported PD (*n* = 20) had more functional limitations and P-E fit problems than matched controls. They also perceived their homes as less usable in relation to activities. These results indicate the importance of studying and comparing such dynamics in sub-groups of the ageing population, but the findings should be replicated in larger samples with a verified PD-diagnosis. In fact, PD-studies that have systematically examined health and housing dynamics are lacking, and older people are often excluded from PD-research [16]. Accordingly, addressing objective as well as perceived aspects of housing and how they are associated with health represents a novel approach for PD-research. The knowledge gained by such studies has potential to nurture the development of more efficient approaches to address individual housing needs, for example, in relocation counseling and housing adaptation services. Moreover, in order to more efficiently address the needs of specific diagnose groups such as people with PD, housing provision at the societal level could benefit from knowledge about housing and health dynamics.

Inspired by the study by Oswald et al. [8], this study aimed to explore associations between aspects of health (e.g., ADL, life satisfaction, depressive symptoms) and objective (i.e., physical environmental barriers) as well as perceived aspects of housing (i.e., usability, meaning of home, external control beliefs) in people with PD.

## Methods

The present study was based on baseline data collected for the project “Home and Health in People Ageing with PD”. The project design, procedure and instrumentation have been described in detail in a study protocol [17]. The previous study by Oswald et al. [8] served as an inspiration for the overall design and selection of variables for the present study. Accordingly, only a subset of the comprehensive data available was utilized.

### Participants

Participants were recruited from three hospitals in the region of Skåne in southern Sweden; 653 individuals fulfilled the inclusion criterion of being diagnosed with PD (G20.9) since at least one year, whereof 158 individuals were not eligible due to the exclusion criteria applied. That is, difficulties in understanding/speaking Swedish and/or extensive cognitive difficulties/other reasons that made the individual unable to give informed consent or take part in the majority of the data collection were used as exclusion criteria. The remaining 437 individuals were invited to participate, whereof 157 declined to participate, 22 were unreachable and two had their diagnosis revised. The final project sample was 255 participants. For the present study, we only included those with complete data on all variables of interest for the study aim. Consequently, 24 (10 men) participants were excluded due to missing data. They were significantly older (*p* = 0.009) and had more severe PD (*p* = 0.011) than the included participants, but their PD-duration did not differ (*p* = 0.722). The final sample for the present study included 231 (62 % men) participants with a mean age of 75 (min-max, 45-93) years.

### Data collection and instruments

The data collection for the project included a self-administered postal survey and a subsequent home visit where structured interviews, observations and clinical assessments were administered. The data collection was administered and performed by two project assistants (experienced reg. occupational therapists) that underwent project-specific training.

Descriptive participant characteristic information (see Table 1) included age, sex, living situation (living alone or not), type of housing and years lived in the present dwelling. Another question targeted financial satisfaction (“All in all, how satisfied are you with the financial situation of your household?”); the scoring can range from 0 (very unsatisfied) to 10 (very satisfied). To describe the severity of PD (in the “on condition”), the Hoehn & Yahr staging scale was used (score range I to V, higher = worse) [18]. The severity of motor symptoms was assessed according to the Unified Parkinson’s Disease Rating Scale (UPDRS, part III); the total score can range from 0 to108 (higher = worse) [19]. The Nonmotor Symptoms Questionnaire (NMSQuest) [20] was used to describe the total number of self-reported non-motor symptoms (30 dichotomous Yes/No items). Cognitive functioning was assessed with the Montreal Cognitive Assessment (MoCA) (max. score = 30, higher = better) [21].

**Table 1:** Participant characteristics, *n* = 231

Variable	Median, q1-q3 (unless otherwise stated)
Age (years), mean (SD)	70 (9.1)
Sex (men), *n* (%)	144 (62)
Living alone (yes), *n* (%)	58 (25)
Years lived in the present home	17, 5-33
Type of housing (apartment/private house)^a^, *n* (%)	101 (44)/ 129 (56)
Financial satisfaction	8, 5–9
PD-duration (years)	8, 5–13
PD-severity (Hoehn & Yahr)	2, 2–3
Severity of motor symptoms (UPDRS part III)	29, 21–39
Cognitive function (MoCA)	26, 23–28
Non-motor symptoms (NMSQuest, total number reported)	10, 6–14

*MoCA* the Montreal Cognitive Assessment (maximum score = 30; higher = better), *NMSQuest* Nonmotor Symptoms Questionnaire (scoring 0–30; higher = worse), *PD* Parkinson’s disease, *UPDRS III* Unified Parkinson’s Disease Rating Scale, motor examination (scoring 0–108; higher = worse), Hoehn & Yahr staging scale (scoring I–V; higher = worse), Financial satisfaction scoring 0–10 (higher = better)

^a^One additional participant lived in special (assisted) housing

The instruments used to cover aspects of health and housing are described in more detail (i.e., number of items, scoring range, interpretation) in Tables 2 and 3, including also descriptive data.

**Table 2:** Descriptives of aspects of health among people with Parkinson’s disease, *n* = 231

		Total score		
Variable, Instrument	Number of items	Possible total scoring range, min-max	Median (q1-q3) unless other is stated	min-max
Functional limitations, HE	12	0–12	4 (2–5)	0–9
Activities of Daily Living, PADLS	1	1–5	2 (2–3)	1–5
Depressive symptoms, GDS-15	15	0–15	2 (1–4)	0–15
Autonomy, PWQ, mean (SD)	9	1–5	3.6 (0.45)	2.3–4.9
Purpose in life, PWQ, mean (SD)	9	1–5	3.3 (0.54)	2.1–4.8
Life satisfaction, item 1 LiSat-11	1	1–6	5 (4–5)	1–6

Decimals are only given if the value is between two categories; results are rounded as to one decimal or two meaningful digits

*HE* the Housing Enabler (a higher value/score = more functional limitations), *GDS-15* the Geriatric Depression Scale (higher = more depressive symptoms), *LiSat-11* the Life Satisfaction Questionnaire (higher=”better”), *PADLS* the Parkinson's disease Activities of Daily Living Scale (higher = more severe difficulties/dependence), *PWQ* Psychological Wellbeing Questionnaire (higher=”better”)

**Table 3:** Decriptives of physical environment barriers and perceived aspects of home among people with Parkinson’s disease, *n* = 231

		Total score		
Variable, Instrument	Number of items	Possible total scoring range, min-max	Median (q1-q3)	min-max
Environmental barriers, HE	161	0–161	67 (59–73)	32–86
Usability in the home, UIMH				
Physical environmental aspects	6	1–5	4.5 (4–5)	1.8–5
Activity aspects	4	1–5	4.8 (4–5)	1–5
Meaning of home, MOH				
Behavioral aspects	6	0–10	7.7 (6.7–9)	2–10
Cognitive emotional aspects	10	0–10	8 (7.2–8.8)	4.5–10
Housing related external control beliefs, HCQ				
Combined (Subscales: powerful others + chance)	16	1–5	2.4 (2–3)	1–4.4

Decimals are only given if the value is between two categories

*HE* Housing Enabler (higher scores = more physical environmental barriers), *UIMH* Usability in My Home Questionnaire (higher scores = higher usability), *MOH* Meaning of Home Questionnaire (higher scores = stronger bonding/attachment to the home), *HCQ* Housing-related Control Beliefs Questionnaire (higher scores = more external control, i.e., “some other person, luck, chance or fate is perceived as explanatory factors for what happens”)

#### Aspects of health

Prior to the home visit the self-administered Parkinson’s Disease Activities of Daily Living Scale (PADLS) [22] was distributed by post; it targets difficulties and dependence in relation to ADL. At the home visit, interview and observation of functional limitations was administered according to the personal component of the Housing Enabler instrument (HE; further described under “Aspects of Housing”) [23]. The following 12 functional limitations were scored as Present/Not present: difficulty in interpreting information; visual impairment; blindness; loss of hearing; poor balance; incoordination; limitations of stamina; difficulties in moving head; reduced upper extremity function; reduced fine motor skill; loss of upper extremity skills; reduced spine and/or lower extremity function.

The “Autonomy” and “Purpose in life” subscales of the Psychological Wellbeing Questionnaire (PWQ) [24] were used. Life satisfaction was evaluated with item 1 of the Life Satisfaction Questionnaire (LiSat-11) [25]. Depressive symptoms were assessed with the Geriatric Depression Scale (GDS-15) [26].

#### Aspects of housing

Objective housing was operationalized as the total number of physical environmental barriers according to the HE [23]. Based on observation during home visits 161 environmental barriers in the home and the immediate outdoor environment were objectively assessed (present or absent) according to national standards for housing design.

Perceived aspects of housing were captured by three interview-administered instruments: the Usability in My Home Questionnaire (UIMH) [27], Meaning of Home Questionnaire (MOH) [9] and Housing-related Control Beliefs Questionnaire (HCQ) [28]. The “Activity aspects” and “Physical environmental aspects” sub-scales of the UIMH were used, as were the “Behavioral” and “Emotional” sub-scales of the MOH. Meaning of home refers to how an individual reacts to and feels about his/her home, whereas external control in relation to the home means that “some other person, luck, chance or fate is perceived as explanatory factors for what happens” [9]. In order to capture external housing control beliefs, the subscales “Powerful others” and “Chance” from the HCQ were combined as previously recommended [29].

### Statistical methods and analysis

To study the association between aspects of health and housing, canonical correlation [30–32] was applied. This method is suitable for exploring relationships between two sets of variables, in order to identify combinations of variables in one of the sets that correlate with combinations of the variables in the other. Both sets of variables are treated equally without any assumptions of cause and effect between them.

The first step of the analysis identified the linear combinations of the variables in each of the two sets that maximise the correlation between them. These combinations are called the first pair of canonical variates, and the correlation between them is denoted the first canonical correlation. Thereafter a second pair of canonical variates, orthogonal to the first pair, was computed. More pairs of canonical variates were computed, but we report only those canonical variates that were statistically significant (*P*-values below 0.05).

For each variable and each significant pair of canonical variates the standardized coefficient and the loading are presented, and the sign of them shows the direction of the association. The loading is the correlation between a variable and the canonical variate; a higher loading (irrespective of the sign) means that the variable is more important. The cut-off used to identify essential variables is an absolute loading above 0.35 [32], corresponding to an explained variance of about 10 %. The use of standardized coefficients make variables measured in different units comparable. The reported square of the canonical correlation (canonical R^2^) represents the overlapping variance of the two canonical variates in a pair. We also present the amount of variance a canonical variate explain from its own set of variables, (computed as the sum of the squared loadings divided by the number of variables) and a redundancy measure, which describes the amount of variance a variate from one of the sets extracts from the other (redundancy = canonical R^2^ × the average of the squared loadings).

#### Sample size

An exact sample size calculation for canonical correlation is infeasible to perform, but previous simulation studies indicate that the most important feature is the ratio between the number of individuals and the number of variables; they recommend that this ratio should be above 42 to interpret the first two pairs of canonical variates and at least around 20 to interpret only the first [31]. We choose to include 12 variables (6 in each set), which corresponds to about 20 individuals per variable, see Tables 2 and 3. This is then considered enough to accurately interpret the largest (i.e., first) canonical correlation, while the remaining canonical correlations should be interpreted more carefully.

The statistical analysis was generated using SAS software, version 6.1 of the SAS Enterprise Guide (Copyright © 2013 SAS Institute Inc). SAS and all other SAS Institute Inc. product or service names are registered trademarks or trademarks of the SAS Institute Inc., Cary, NC, USA.

## Results

The analysis between the health variables and housing variables set yielded two significant pair of variates with the canonical correlations 0.68 (*p* < 0.0001) and 0.33 (*p* = 0.0112), respectively. For details, see Table 4.

**Table 4:** Canonical correlations: Relations of aspects of health and aspects of housing among people with PD, *n* = 231

	First canonical variate	Second canonical variate
Canonical correlation	0.68, *p* < 0.0001	0.33, *p* = 0.0112
Canonical R^a^	0.46	0.11
Aspects of health	Loadings / stnd.can.coeff.	Loadings / stnd.can.coeff.
Functional limitations (HE)^b^	**0.79** / 0.32	0.21 / 0.45
Activities of Daily Living (PADLS)^b^	**0.84** / 0.45	−0.05 / −0.26
Depressive symptoms (GDS-15)^b^	**0.65** / 0.14	−0.12 / −0.08
Autonomy (PWQ)^a^	**−0.53** / −0.23	0.10 / 0.03
Purpose in life (PWQ)^a^	**−0.56** / −0.14	**−0.50** / −0.80
Life satisfaction (LiSat-11)^a^	**−0.59** / −0.14	**0.56** / 0.86
Percent of variance (average of squared loadings)	0.45	0.11
Redundancy	0.21	0.01
Aspects of housing		
Physical environmental barriers (HE)^b^	−0.19 / −0.29	−0.21 / −0.17
Usability- Activity aspects (UIMH)^a^	**−0.49** / −0.16	**0.37** / 0.23
Usability- Physical environmental aspects (UIMH)^a^	**−0.58** / −0.16	**0.38** / 0.05
Meaning of home- Behavioral aspects (MOH)^c^	**−0.72** / −0.55	**0.51** / 0.17
Meaning of home- Cognitive emotional aspects (MOH)^c^	−0.28 / 0.16	**0.82** / 0.68
External housing related control beliefs (HCQ)^d^	**0.77** / 0.54	**0.39** / 0.55
Percent of variance (average of squared loadings) Redundancy	0.30 0.14	0.23 0.03

Loadings above the cut-off value (0.35) are bolded. *NB* The second canonical variate was significant but should be interpreted with caution due to the small sample size and small degree of explained variance

*HCQ* Housing-related Control Beliefs Questionnaire, *HE* Housing Enabler, *GDS-15* Geriatric Depression Scale, *LiSat-11 (item1)* Life Satisfaction Questionnaire, *MOH* Meaning of Home Questionnaire, *PADLS* Parkinson’s Disease (PD) Activities of Daily Living Scale, *PWQ* Psychological Wellbeing Questionnaire, *UIMH* Usability in My Home Questionnaire

^a^Higher scores = better

^b^Higher scores = worse

^c^Higher scores = mirror a stronger bonding/attachment to the home

^d^Higher scores = more external control

For the first pair of variates the canonical R^2^ was 0.46. In the health variable set the separate loadings showed that ADL difficulties/dependence (PADLS) and functional limitations (personal component of HE) contributed the most, but all variables loaded above the 0.35 cut-off. ADL difficulties/dependence and functional limitations tended to have higher standardized canonical coefficients while depressive symptoms (GDS-15), autonomy and purpose of life (PWQ) and life satisfaction (item 1, LiSat-11) scored lower in this respect. This indicates an internal correlation between these four variables and functional limitations and/or the ADL variable. In the housing set, the variables with the strongest loadings were external control beliefs (HCQ) and behavioral aspects of meaning of home (MOH). In addition to having lower loadings, the two usability subscales (UIMH) scored lower also in terms of standardized canonical coefficients. Physical environmental barriers (environmental component of HE) and cognitive emotional aspects of meaning of home (MOH) did not reach the 0.35 cut-off value for loadings. To summarize, difficulties/dependence in ADL and having more functional limitations related to perceived aspects of housing in terms of higher external control and lower perceived meaning of home but not to the objective aspect physical environmental barriers.

Although a significant relationship was found for the second canonical correlation, the shared variance between the health and housing variates was considerably lower; R^2^ = 0.11. Among the health variables, life satisfaction and purpose in life had the highest loadings and were the only variables that exceeded the cut-off value. Loadings > 0.35 were seen for all the perceived aspects of housing variables but not for the objective aspect (physical environmental barriers). The two variables with the highest loadings were cognitive-emotional and behavioral aspects of meaning of home, where the former showed the highest standardized coefficient.

## Discussion

The main contribution of this study is the demonstration of patterns of relationships between health and housing among people with PD, showing that there is a significant relationship between aspects of health and perceived aspects of housing. The results show that external control beliefs and behavioral aspects of meaning of home contribute the most to the housing variate, whereas ADL difficulties/dependence and functional limitations contribute the most to the health variate. The first canonical correlation suggests that participants that have difficulties/dependence in ADL and have more functional limitations tend to rely on external influences managing their housing situation and perceive their homes as less meaningful from a behavioral perspective. To the best of our knowledge, this is the first study that elucidates this kind of relations in this sub-group of the ageing population, and in a detailed manner. Thus, the study adds to the understanding of the complex dynamics of health and housing among people ageing with a chronic and progressive disease.

Some of the present findings are in line with the results of Oswald et al. [8], but there are also differences. Regarding similarities, difficulties and dependence in ADL was among the variables that contributed the most to the variate in the health variable set in both studies. Given the well-known fact that ADL dependence increases with age [1], this was an expected finding when studying general population samples of very old people (Oswald et al. [8]). That ADL played an important role also in the present study is not surprising either since ADL is affected early on after having received a PD-diagnosis and in mild PD-stages [10, 11]. In a nursing home setting, people with PD have also shown a faster rate of functional decline than other residents [33]. That difficulties and dependence in ADL as well as functional limitations contribute the most to the health variate in both studies underlines the importance of addressing these aspects in general population samples of very old people as well as among people with PD.

Another similarity between the present study and the one by Oswald et al. [8] is that an objective aspect of housing such as the total number of physical environmental barriers did not contribute significantly to the housing variable set. The fact that perceived aspects of housing did contribute may be of importance for the development of person-centered care in the home setting. This facet of our results suggests that perceived aspects of housing should be considered when targeting housing issues in PD-care and rehabilitation. This is important as in current practices mainly objective aspects of housing are in focus.

Discussing the findings on perceived aspects of housing in more detail, external housing related control beliefs showed the highest loading within the housing variate which indicates that this aspect is specifically important among people with PD. Control beliefs are related to self-efficacy [34], and general self-efficacy has been shown to significantly relate to the severity of PD [35]. Those in the most advanced stages of PD (i.e., Hoehn and Yahr IV–V) reported significantly lower general self-efficacy than those in milder disease stages [35]. However, longitudinal studies are lacking concerning control beliefs as well as general self-efficacy in people ageing with PD; such studies are needed in order to fully understand these relationships. That external housing related control beliefs showed the highest loading is in fact in contrast to the results by Oswald et al. [8], where instead behavioral aspects of meaning of home contributed the most to the housing variate, consistently in five national contexts [8]. Although behavioral aspects of meaning home play a role also among people with PD, the discrepancy might suggest that people ageing with a chronic and progressive neurodegenerative disorder such as PD rely more on external control in relation to housing. That is, this finding indicates that people with PD are more dependent on others when it comes to housing issues. Caregivers of people with PD have furthermore identified depleted informal or formal support as one of the triggers for long-term care placement decisions [36]. Moreover, caregiver burden increases with increased severity of PD especially in the presence of mental health problems (e.g., depression, hallucinations) and falls [37]. If an ADL-dependent person with PD is living in ordinary housing and expresses high housing related control beliefs, it might be of importance to also provide support for the family and caregivers.

Overall in health care and social service practices, interventions related to housing are based on approaches not sufficiently underpinned by scientific evidence. The present findings suggest that people with PD perceive their homes as less meaningful from a behavioral perspective, which points to the need for more awareness of early signs of such associations. In practice, this implies that professionals should be attentive to how people with PD reason regarding meaning of home and whether this changes along the course of the disease. As people age the home becomes more important, and the process of residential reasoning [38] should be supported to help people arrive at well informed choices and decisions regarding individual housing adaptation or a potential move. Most such counselling is based mainly on considerations of physical functioning, while the results of the present study indicate that also perceived aspects of housing are important. Accordingly, the professionals involved should be more attentive also to such aspects and the fact that they are related to health. Ageing research on housing and health dynamics underscores the need to develop more efficient housing counseling services to help older people deal with ambivalence, fears, worries, and practical considerations about their housing situation [39]. The present study contributes to the development of such knowledge for people ageing with a specific diagnose such as PD.

Some methodological considerations need to be acknowledged. We used canonical correlations with data that to a major extent were ordinal and not all variables did actually fulfil the normal distribution criterion. Still, in the absence of non-parametric alternatives to study correlation between sets of variables, the use of canonical correlation made it possible to study a complexity otherwise left unexplored. As a sensitivity analysis, we dichotomized the variables that did not fulfil the normal distribution criterion and the results did not change (data available on request). Accordingly, we consider the results presented as valid. Although inspired by the study by Oswald et al. [8], we did not include identical variables or used identical instruments in all instances. For example, we do not have data on positive and negative affect in the database used and ADL was not assessed in an identical manner. Moreover, housing satisfaction was not included in the present study due to lack of variance and a very prominent ceiling effect (77 % scored at maximum, data available on request). Moreover, it should be noted that Oswald et al. [8] found that the magnitude of accessibility problems (total score of HE) was of importance. However, as assessed with the HE accessibility problems is a function of the profile of functional limitations of the individual and the physical environmental barriers present in the dwelling and the closest exterior surroundings [23]. Since recent research has shown that functional limitations contribute the most to the magnitude of accessibility problems [15], despite that we have access to the variable accessibility problems it was not included in our analyses. That is, for conceptual stringency and to avoid overlap between the two sets of variables used in the canonical correlation we considered it more valid to include the number of functional limitations in the health variable set and number of physical environmental barriers in the housing variable set.

When studying associations between different health and housing variables it is obvious that the result depends on the variables included. While not specifically addressed in the present study, it might be that cognitive status, in itself or indirectly via other health variables, might influence perceived housing. As cognition is known to be affected in PD, this is a topic that deserves attention in future studies. Moreover, the challenge of understanding whether motor symptoms and/or cognitive status are the source for ADL difficulties in people with PD was recently highlighted [40]. It should be noted that several additional health variables may play a role in PD, such as low self-efficacy, PD-specific motor features and additional non-motor symptoms besides depression. However, the present sample size did not allow for including more variables when using canonical correlations. Importantly, although the second canonical variate was significant, it should be interpreted with caution due to the small sample size and small degree of explained variance.

## Conclusions

This study suggests that people with PD who have more functional limitations, difficulties and dependence in ADL perceive their homes as less meaningful from a behavioral perspective and tend to rely on external influences managing their housing situation. The results indicate that there might be differences between people with PD as compared to general population samples, but more research is certainly needed to deepen the knowledge in this new area of inquiry. This kind of knowledge poses health care and social services professionals in a better position to efficiently address problems related to housing and health among people with PD.
